# Development and internal validation of risk stratification tool for lymph node metastasis in pT3-4 laryngeal squamous cell carcinoma patients

**DOI:** 10.1016/j.bjorl.2024.101535

**Published:** 2024-11-18

**Authors:** Changding He, Yu Heng, Xiaoke Zhu, Jian Zhou, Lei Tao

**Affiliations:** Fudan University, Department of Otorhinolaryngology, Eye and ENT Hospital, Shanghai, China

**Keywords:** Laryngeal squamous cell carcinoma, Lymph node metastasis, Risk stratification

## Abstract

**Objective:**

To identify risk factors for Lymph Node Metastasis (LNM) in pT3-4 Laryngeal Squamous Cell Carcinoma (LSCC) patients with negative margins, and develop a nomogram to predict LNM risk.

**Methods:**

872 patients were divided into training (2010–2014) and validation (2015–2016) cohorts. Univariate and multivariate analyses identified LNM risk factors. A nomogram incorporating significant factors was developed in the training cohort.

**Results:**

Smoking history, maximal tumor diameter ≥3.0 cm, depth of tumor invasion >1.0 cm, and supraglottic tumor location were significantly associated with LNM on multivariate analysis. A predictive nomogram incorporating these factors showed good discrimination (C-index > 0.7) in both cohorts. Patients were stratified into low, moderate and high-risk subgroups based on total risk scores.

**Conclusions:**

A LNM risk prediction model and risk grouping system was established, which may aid treatment selection for pT3-4 LSCC patients. The model and algorithm could help optimize neck management for this high-risk patient population.

**Level of evidence:**

2.

## Highlights

• Four factors identified predicting lymph node metastasis in laryngeal carcinoma.
• Nomogram created incorporating factors, C-index >0.75 proves high accuracy.
• Patients stratified into low/intermediate/high risk groups by nomogram.
• Lymph node metastasis rates significantly differ among risk groups.
• Model provides guidance for individualized treatment in laryngeal carcinoma.

## Introduction

Laryngeal carcinoma, specifically squamous cell carcinomas, is a prevalent malignant tumor in the head and neck region in China. The incidence rates per year indicate a higher prevalence in men compared to women, with a male to female ratio of approximately 7–9 to 1.^1^ Laryngeal Squamous Cell Carcinomas (LSCC) is classified into initial and advanced stages, occurring in the supraglottic, glottic, and subglottic regions. Therapeutic options vary depending on the stage and subsite of the tumor. Advanced stage LSCC has a bleak prognosis.^2^ Compared to initial disease, locally advanced LSCC has significantly lower survival rates and larynx preservation rates. In particular, supraglottic LSCC has a relatively poorer prognosis compared to glottic LSCC.^3^ Unfortunately, there has been limited progress in improving survival trends for LSCC over the past few decades.^4^ Numerous risk factors have been implicated in the etiology of laryngeal carcinoma, among which tobacco and alcohol consumption are of paramount significance. The association between tobacco use and the incidence of laryngeal cancer has been established to exhibit a linear pattern, with smokers exhibiting a 10 to 15-fold elevated risk compared to non-smokers.^5^, ^6^

For early-stage LSCC, the primary organ preservation therapies are radiotherapy and endoscopic surgery. With the incorporation of concurrent chemoradiotherapy and neoadjuvant therapy, the treatment options for advanced stage LSCC have become more diversified and are now standardized according to AJCC guidelines. However, total or partial laryngectomy remains the primary standard of care for advanced T3-T4 stage disease. In formulating individualized treatment strategies, we first perform a preliminary Lymph Node Metastasis (LNM) risk assessment based on factors such as primary tumor location and staging. We also utilize CT, MRI and other imaging tools to initially evaluate the status of cervical lymph nodes. For patients with imaging-negative lymph nodes but high risk of occult metastasis, we would consider prophylactic dissection of the relevant nodal regions. However, Lymph Node Dissection (LND) can also lead to complications including bleeding, infection, shoulder dysfunction, as well as cosmetic and scar issues, which may severely impact patients’ quality of life.

Therefore, we must weigh the potential benefits and risks when deciding on these treatment modalities. Accurate preoperative prediction of lymph node involvement is crucial for individualized neck management in LSCC patients with advanced disease. This study aims to identify risk factors for neck LNM in patients with locally advanced (pT3-4) LSCC, including those with clinically Negative neck (cN-) and known neck involvement (cN+). By comprehensively evaluating all patient groups, we aim to develop a risk stratification tool applicable to various clinical environments and validate its effectiveness and stability across different subgroups.

## Methods

### Patients cohort

A retrospective study was conducted of patients with pT3-4 LSCC who underwent surgery from 2010 to 2014 and from 2015 to 2016 at the Fudan University, Department of Otorhinolaryngology, Eye & ENT Hospital. The study was approved by the Clinical Medical Research Ethics Committee of the Fudan University, Department of Otorhinolaryngology, Eye & ENT Hospital. Written informed consent was obtained from all participants or their authorized representatives prior to study enrollment. Patients were included if they had: (1) Glottic or supraglottic LSCC; (2) A definitive diagnosis; and (3) A negative surgical margin pathologically. Patients were excluded if they had: (1) Multiple primary tumors; (2) Distant metastases; (3) Preoperative neoadjuvant therapy (4) Prior radiotherapy or salvage surgery before operative treatment. A total of 872 patients with primary pT3-4 LSCC treated with surgery were enrolled. Of these, 665 patients treated from 2010 to 2014 comprised the training cohort and 207 patients treated from 2015 to 2016 comprised the validation cohort.

### Data

Demographic and clinical characteristics including age, gender, smoking history, alcohol consumption, hypertension, diabetes history and TNM stage were collected. Postoperative information such as tumor differentiation, margin status, Maximum Tumor Diameter (MTD) and depth of infiltration were also documented in our retrospective database. All patients underwent primary tumor resection. For T3 glottic LSCC, if cervical LNM was detected on preoperative imaging, we performed unilateral functional neck dissection. In cases without LNM on preoperative imaging, neck dissection was typically not performed. For T3 Supraglottic type: We routinely performed unilateral or bilateral functional neck dissection, depending on whether the primary lesion crossed the midline and location of positive lymph nodes for glottic LSCC cases without neck dissection, we closely monitored for lymph node recurrence during follow-up. Cases without recurrence within 6-months were classified into the Non-LNM group, while those with recurrence were considered to have occult metastasis and were classified into the LNM group. For T4 LSCC, we routinely performed bilateral functional neck dissection. Radical neck dissection was conducted if there was jugular vein involvement.

### Statistical analysis

Patients were divided into training and validation sets according to admission time. SPSS 24.0 was used for data analysis. Univariate Cox proportional hazard models were used to initially screen for potential predictors (*p* < 0.15). All clinically significant variables were then included in the multivariate Cox model. The Hazard Ratio (HR) with 95% Confidence Interval (95% CI) was selected as the effect size in univariate and multivariate analyses. Significant predictors in multivariate analysis were used to create the nomogram. Internal and external validation were performed using the training and validation sets respectively. The risk prediction model was generated in R 3.5.1. Receiver operating characteristic curves were plotted, and the Concordance index (C-index) calculated to evaluate model discrimination. Calibration curves assessed model calibration. Analyses were performed using SPSS 25.0, R 3.5.1 and GraphPad Prism 9. Statistical significance was defined as *p* < 0.05. To determine the optimal cutoff values for tumor diameter and invasion depth, we used ROC curve analysis. A tumor diameter of 3cm and an invasion depth of 1 cm had the highest AUC, indicating high sensitivity and specificity. Thus, we selected 3 cm and 1 cm as the respective cutoffs.

## Results

### Demographics, clinical characteristics, and LNM of all patients

In this study, a total of 872 patients with LSCC were enrolled and divided into a training set of 665 patients and a validation set of 207 patients. All patients underwent surgical resection of the primary tumor, with or without concurrent LND. Based on the postoperative pathological reports, patients were further classified into the Non-Lymph Node Metastasis (Non-LNM) group or the LNM group.

Table 1 presents the distribution of patient characteristics in each group, including age, gender, smoking and drinking history, cancer staging, maximum tumor diameter, depth of invasion, tumor sublocation, and histopathological differentiation. Our analysis revealed significant statistical differences between Non-LNM and LNM patients in both training and validation sets regarding maximum tumor diameter (≥3 cm), depth of invasion (>1 cm), and tumor subregion (*p* < 0.05). This suggests these factors are closely associated with LNM in LSCC.

**Table 1:** The clinicopathological characteristics of pT3-4 LSCC patients.

	Training Group							Validation Group						
	All Patients		Non-LNM Group		LNM Group		p-value	All Patients		Non-LNM Group		LNM Group		p-value
	n = 665	%	n = 494	%	n = 171	%		n = 207	%	n = 156	%	n = 51	%	
**Age**							0.239							0.315
≤60	333	50.1	254	51.4	79	46.2		106	51.2	83	53.2	23	45.1	
>60	332	49.9	240	48.6	92	53.8		101	48.8	73	46.8	28	54.9	
**Gender**							0.071							0.196
Female	16	2.4	15	3.0	1	0.6		5	2.4	5	3.2	0	0.0	
Male	649	97.6	479	97.0	170	99.4		202	97.6	151	96.8	51	100.0	
**History of smoking**							0.018							0.030
No	178	26.8	144	29.1	34	19.9		66	31.9	56	35.9	10	19.6	
Yes	487	73.2	350	70.9	137	80.1		141	68.1	100	64.1	41	80.4	
**History of alcoholism**							0.091							0.955
No	352	52.9	271	54.9	81	47.4		117	56.5	88	56.4	29	56.9	
Yes	313	47.1	223	45.1	90	52.6		90	43.5	68	43.6	22	43.1	
**pT stage**							0.891							0.123
pT3	513	77.1	380	76.9	133	77.8		149	72.0	108	69.2	41	80.4	
pT4	152	22.9	114	23.1	38	22.2		58	28.0	48	30.8	10	19.6	
**Maximum tumor diameter (MTD)**							<0.001							0.025
<3.0 cm	342	51.4	298	60.3	44	25.7		93	44.9	77	49.4	16	31.4	
≥3.0 cm	323	48.6	196	39.7	127	74.3		114	55.1	79	50.6	35	68.6	
**Depth of tumor invasion**							<0.001							0.039
≤1.0 cm	236	35.5	215	43.5	21	12.3		99	47.8	81	51.9	18	35.3	
>1.0 cm	429	64.5	279	56.5	150	87.7		108	52.2	75	48.1	33	64.7	
**Subregion**							<0.001							<0.001
Supraglottic	253	38.0	135	27.3	118	69.0		89	43.0	54	34.6	91	68.6	
Glottic	412	62.0	359	72.7	53	31.0		118	57.0	102	65.4	37	31.4	
**Differentiation**							0.086							0.401
High	294	44.2	228	46.2	66	38.6		95	45.9	69	44.2	26	51.0	
Moderate / low	371	55.8	266	53.8	105	61.4		112	54.1	87	55.8	25	49.0	

Preoperative imaging assessed 252 patients as Node-positive (N+) and 620 as Node-negative (N-), a 28.9% positive rate. Postoperative pathology confirmed 222 N + and 650 N- patients, a 25.5% positive rate (Supplementary Table S1), indicating discrepancy between preoperative and postoperative lymph node assessment. Preoperative imaging demonstrated 95.5% sensitivity and 93.8% specificity versus postoperative pathology, with some false positives/negatives. The McNemar test comparing preoperative and postoperative assessment showed statistically significant difference (χ^2^ = 18, *p* < 0.05).

### Development and validation of the risk-scoring model

Using data from the training cohort, high-risk factors for LNM were identified through univariate and multivariate Cox regression analyses, as shown in Table 2. Both univariate and multivariate analyses consistently demonstrated that smoking history, MTD, depth of invasion, and tumor sublocation were significantly associated with the occurrence of LNM (*p* < 0.05).

**Table 2:** Univariate and multivariate analyses of LNM for pT3-4 LSCC patients.

	Univariate analysis		Multivariate analysis	
Factors	Hazard ratio (95% CI)	p-value	Hazard ratio (95% CI)	p-value
Age		0.240		
>60 vs. ≤60	1.232 (0.870–1.747)			
Gender		0.107		
Male vs. Female	5.324 (0.698–40.607)			
History of alcoholism		0.091		
Yes vs. No	1.350 (0.953–1.913)			
Differentiation		0.087		
Moderate/Low vs. High	1.364 (0.956–1.945)			
History of smoking		0.019		0.040
Yes vs. No	1.658 (1.086–2.530)		1.629 (1.021–2.597)	
MTD		<0.001		<0.001
≥3.0 cm vs. <3.0 cm	4.388 (2.979–6.465)		2.567 (1.649–3.998)	
Depth of tumor invasion		<0.001		0.003
>1.0 cm vs. ≤1.0 cm	5.504 (3.372–8.986)		2.339 (1.340–4.085)	
Subregion		<0.001		<0.001
Glottic vs. Supraglottic	0.169 (0.116‒0.247)		0.244 (0.163‒0.365)	

**Fig. 1:**
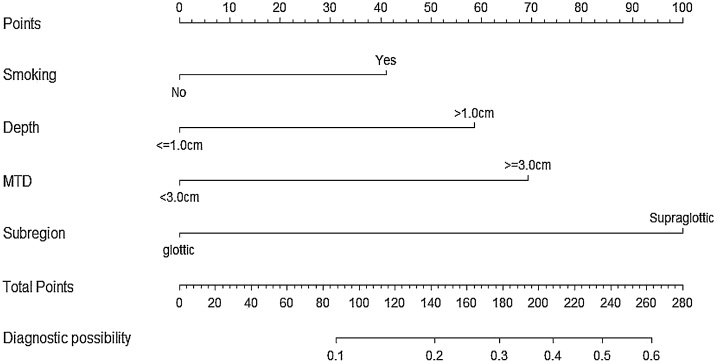
The length of the line for each item represents the possibility of LNM. MTD, Maximum Tumor Diameter.

**Fig. 2:**
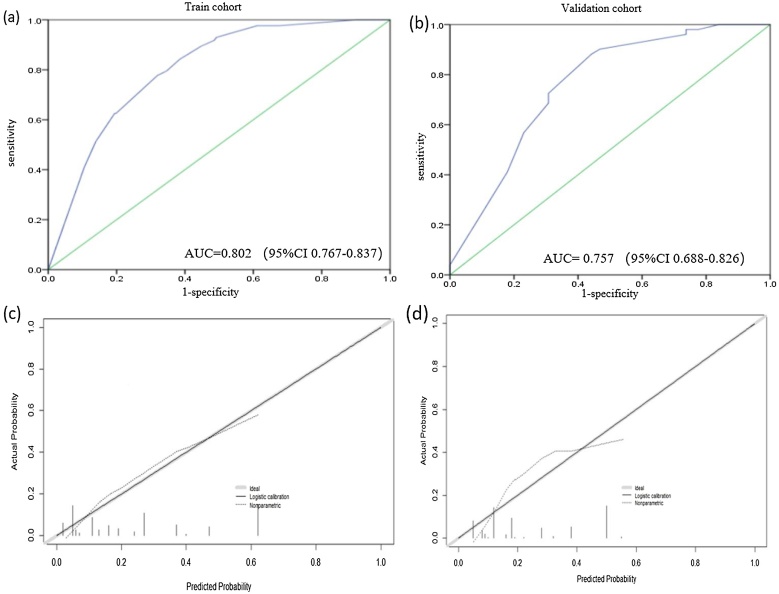
ROC curves of the nomogram. (a) ROC curve and AUC of the nomogram in the training cohort. (b) ROC curve and AUC of the nomogram in the validation cohort. (c) Calibration curve of the nomogram for predicting LNM risk. (d) Calibration curve of external validation in the validation cohort.

Based on these four risk factors, a predictive nomogram was developed as illustrated in Fig. 1. This nomogram can be utilized to estimate the probability of cervical LNM in patients with stage T3-T4 LSCC. Each risk factor is proportionately represented by the scale of the corresponding line in the nomogram, reflecting its relative contribution to the risk of lymph node involvement. By aggregating the scores of all four factors, individual risk for LNM can be calculated. We evaluated the predictive performance of this nomogram using C-index and calibration curves through internal and external validation. The C-indexes were 0.802 (95% CI: 0.767‒0.837) and 0.757 (95% CI: 0.688‒0.826) for the training and validation cohorts, respectively (Fig. 2a and b). The nomogram predictions were highly consistent with patients ‘actual outcomes, demonstrating strong predictive power and discrimination. Additionally, the calibration curves (Fig. 2c and d) revealed good agreement between nomogram-predicted probability of LNM and actual observed probability.

### Risk stratification for LNM of LSCC patients with pT3-4 tumors

**Fig. 3:**
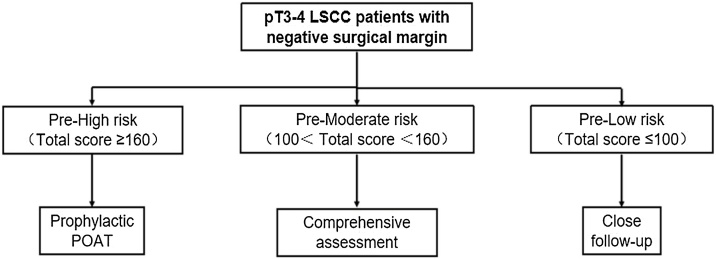
Risk stratification and postoperative adjuvant radiotherapy strategy for LSCC patients. PORT, Postoperative Radiotherapy.

Using the nomogram model, we stratified the risk of cervical LNM in patients from the training and validation cohorts (Fig. 3). By assessing the distribution of total scores, we determined cutoff values to categorize T3-T4 stage laryngeal cancer patients into low-risk (total score ≤100; n = 196), intermediate-risk (total score 100–160; n = 178), and high-risk (total score ≥160; n = 291) groups. In the training cohort, the three risk groups showed significantly different rates of lymph node positivity, 1.5%, 19.1%, and 46.0%, respectively (*p* < 0.001). Similarly, in the validation cohort, the rates were 3.9%, 23.7%, and 47.2% for the low, intermediate, and high-risk groups, respectively, also reaching significance (*p* < 0.001) (Table 3).

**Table 3:** Risk stratification for LNM of LSCC patients with pT3-4 tumors.

	Training Group (n = 665)				Validation Group (n = 207)			
	Low risk (n = 196, %)	Moderate risk (n = 178, %)	High risk (n = 291, %)	*p*-value	Low risk (n = 76, %)	Moderate risk (n = 59, %)	High risk (n = 72, %)	p-value
Negative LNM	193 (98.5)	144 (80.9)	157 (54.0)	<0.001	73 (96.1)	45 (76.3)	38 (52.8)	<0.001
Positive LNM	3 (1.5)	34 (19.1)	134 (46.0)		3 (3.9)	14 (23.7)	34 (47.2)	

### Univariate and multivariate analyses of contralateral LNM in pT3-4 LSCC patients

In our cohort of patients afflicted with pT3-4 LSCC, we investigated the prevalence of contralateral LNM ‒ that is, LNM occurring on the opposite side to the original tumor. We applied both univariate and multivariate Cox regression analyses to discern the factors influencing this phenomenon. The univariate analysis indicated a significant correlation between contralateral LNM and several variables: smoking status, an increase in MTD, a greater depth of tumor invasion, and specific tumor subregions (all *p*-values < 0.05). Subsequent multivariate analysis, however, distilled these down to two independent predictors. An increased MTD emerged as a factor (Hazard Ratio [HR = 1.2], 95% Confidence Interval [95% CI 1.1–1.4]), as well as the tumor subregion (HR = 2.1, 95% CI 1.4–3.1), as detailed in Table 4.

**Table 4:** Univariate and multivariate analyses of contralateral LNM for pT3-4 LSCC patients.

Factors	Hazard ratio (95% CI)	p-value	Hazard ratio (95% CI)	p-value
Age		0.742		
>60 vs. ≤60	1.111 (0.594–2.076)			
Histor y of alcoholism		0.573		
Yes vs. No	0.834 (0.444–1.567)			
Differentiation		0.078		
Moderate/Low vs. High	1.833 (0.935–3.592)			
History of smoking		0.031		0.051
Yes vs. No	2.845 (1.100–7.357)		2.627 (0.995–6.937)	
MTD		<0.001		0.003
≥3.0 cm vs. <3.0 cm	5.816 (2.545–13.293)		4.178 (1.641–10.637)	
Depth of tumor invasion		0.005		0.937
>1.0 cm vs. ≤1.0 cm	3.511 (1.457–8.461)		0.959 (0.339–2.712)	
Subregion		<0.001		<0.001
Glottic vs. Supraglottic	0.149 (0.070‒0.317)		0.207 (0.093‒0.460)	

## Discussion

LSCC arises most commonly in the glottis (69%), followed by the supraglottis (30%) and subglottis (1%). Compared to glottic LSCC, supraglottic tumors have a higher risk of LNM, attributed to richer lymphatic drainage and poorer differentiation.^7^, ^8^ LNM is a major prognostic factor for LSCC survival, potentially impacting initial treatment effectiveness and recurrence.^9^ Several clinical examinations detect cervical LNM, including Computed Tomography (CT), Magnetic Resonance Imaging (MRI), ultrasound, and Positron Emission Tomography (PET).^10^ However, despite negative preoperative imaging, LNM still occurs in some clinically node-negative patients. This is largely due to the modest ∼70% sensitivity of current imaging and inability to detect micrometastases.^11^ While LND for all patients ensures adequate treatment, it may also lead to overtreatment and heightened bleeding/lymphorrhea risks in patients unnecessitating LND. In addition, unnecessary cervical LND should be avoided as it may be a risk factor for pharyngocutaneous fistula after total laryngectomy.^12^ Hence, precise determination of LNM risk is vital for optimizing laryngeal cancer treatment approach and prognosis.

Additionally, clinical staging (cTNM) plays an initial role in cancer management by providing physicians a preliminary understanding of the disease extent and severity, which is key for formulating initial treatment plans. Complementing cTNM is pathological staging (pTNM), which after surgery offers a more precise description of the disease through detailed histological examination, and is crucial for prognosis assessment and designing subsequent treatment strategies. For patients with advanced T3 and T4 laryngeal cancers, accurate preoperative LNM prediction models can greatly impact treatment decisions, aiming to improve prediction accuracy of cervical LNM. Through such models, unnecessary treatment burden can be avoided for patients without metastasis, while more targeted surgical interventions and radiotherapy planning can be delivered to patients with metastatic risks, reducing postoperative complications while optimizing outcomes. Moreover, the model facilitates individualized treatment regimen design. It also assists physicians to implement risk-stratified patient management based on assessments, thereby providing more tailored follow-up and visit plans, which may potentially improve overall patient survival and quality of life.

Logistic regression identified smoking history, tumor size, depth of invasion, and laryngeal cancer subsite as independent risk factors for LNM. A LNM predictive nomogram incorporating these factors was constructed. This model demonstrated excellent predictive performance on internal and external validation, with C-indices exceeding 0.75, indicating high accuracy. The nomogram effectively stratified T3-4 laryngeal cancer patients into low, intermediate, and high-risk groups, with significantly different LNM rates between groups.

This pioneering study developed and validated a clinical nomogram for individualized LNM risk estimation in advanced laryngeal cancer, providing important guidance for treatment decisions. This clinical prediction model has important application value in guiding the graded precision treatment of laryngeal cancer. For low-risk patients, unnecessary cervical LND can be avoided to reduce the risks of bleeding and lymph leakage; while for high-risk patients, thorough cervical LND can ensure adequate tumor treatment.

We acknowledge the differences in lymph node involvement between supraglottic and glottic tumors, which have been well documented in the literature.^3^ However, as our study was designed to identify overall risk factors for LNM in patients with locally advanced LSCC, we chose not to perform a comparative analysis based on lesion location. Additionally, further subdivision could lead to insufficient sample sizes, affecting statistical power. Future research could focus on detailed risk factor analysis for specific tumor locations to provide more nuanced clinical guidance. Limitations of this study include its retrospective design, single-center patient sampling, and restriction to two laryngeal cancer subsites. We indeed used postoperative data (MTD and depth of invasion) to construct our predictive model, rather than relying on preoperative imaging in clinical practice. This decision was made because the sizes obtained from preoperative imaging and postoperative pathology are not always consistent. In future research, we plan to incorporate more preoperative imaging data, evaluate its application in predictive models, and compare it with postoperative pathological results. Future studies should utilize multicenter cohorts with larger sample sizes and refine lymph node classification to enhance model accuracy and applicability.

## Conclusion

This study presents a quantitative risk assessment of LNM in patients with stage T3-T4 LSCC. We developed a predictive model for LNM risk stratification in LSCC patients and designed a clinical algorithm to guide treatment strategy selection and support clinical decision-making. The model and algorithm could help optimize neck management for this high-risk patient population.

## Ethics declarations

The study was approved by the Clinical Medical Research Ethics Committee of the Eye, Ear, Nose and Throat Hospital of Fudan University. All procedures were performed in accordance with the principles of the Declaration of Helsinki. Written informed consent was obtained from all participants or their authorized representatives prior to study enrollment.

## Funding

This work was supported by the National Natural Science Foundation of China, Award Numbers: 81772878, 30801283.

## Conflicts of interest

The authors report no conflicts of interest. The authors are responsible for the content and writing of the article.
