# Supplementary material for: Development and internal validation of risk stratification tool for lymph node metastasis in pT3-4 laryngeal squamous cell carcinoma patients
Source: Braz J Otorhinolaryngol. 2024 Nov 18;91(2):101535. doi: 10.1016/j.bjorl.2024.101535 (PMC11615891; doi:10.1016/j.bjorl.2024.101535)
Supplement: Supplementary file 1 [file mmc1.docx]

**BJORL-D-24-00055_Supplementary Material**

**Supplementary Table 1** Comparison of preoperative imaging and postoperative pathology for LNM in LSCC patients.

|  | **Postoperative N+** | **Postoperative N-** | **Total** |
| --- | --- | --- | --- |
| **Preoperative N+** | 212 | 40 | 252 |
| **Preoperative N-** | 10 | 610 | 620 |
| **Total** | 222 | 650 | 872 |
